# Complete mithochondrial genome of *Ozobranchus jantseanus* (Hirudinida: Arhychobdellida: Ozobranchidae)

**DOI:** 10.1080/23802359.2017.1318684

**Published:** 2017-04-21

**Authors:** Xinhua Liu, Dan Luo, Yuanli Zhao, Qianqian Zhang, Jinyong Zhang

**Affiliations:** a Fish Diseases Laboratory, State Key Laboratory of Freshwater Ecology and Biotechnology, Institute of Hydrobiology, Chinese Academy of Sciences, Wuhan, PR China;; b University of Chinese Academy of Science, Beijing, China

**Keywords:** *Ozobranchus jantseanus*, mitochondrial genome, cryopreservation

## Abstract

The complete mitochondrial genome (14,864 bp) of *Ozobranchus jantseanus* was sequenced and characterized. The genome was constituted of 13 protein-coding genes (PCGs), 2 rRNAs, 22 tRNAs and a non-coding region (NCR). The content of AT was 72.42% and 84.95% for the whole genome and NCR, respectively. The gene order of *O. jansteanus* was identical to those of typical annelids. The phylogenetic analyses constructed by the combined 13 PCGs showed the present species clustered within the Rhynchobdellida clade. This is the first complete mitochondrial genome from the family Ozobranchidae which will provide useful genetic markers for identification, ecological and evolutionary studies of leeches.

*Ozobranchus jantseanus* was originally reported in the body surface of Chinese pond turtle *Chinemys reevesiis* in Wuchung, China (Yang 1996), then it was also recorded in different turtle species of geographically locations in Japan (Yamauchi & Suzuki 2008; Yamauchi et al. 2012). Most interestingly, it was recently reported that *O. jantseanus* could be surviving after long exposure (24 hours) in extremely low temperature (–196 °C) without any acclimation period of pretreatment (Suzuki et al. 2014). So, elucidating the surprisingly tolerance mechanism of this ozobranchid to freezing will be definitely interested for future studies of cryopreservation. Here, the complete mitochondrial genome (mitogenome) of *O*. *jantseanus* was obtained by long PCR and Sanger sequencing and fully annotated. The samples were collected in Jieyang city, Guangdong province, Southern China. The voucher specimen (NO. MTR20161219) was deposited in the Institute of Hydrobiology, Chinese Academy of Sciences.

The complete mitogenome of *O. jantseanus* was 14,864bp in length deposited in GenBank with accession number KY861060, contains 13 protein-coding genes (PCGs), 22 tRNAs, 2 rRNAs and 1 major non-coding control region (NCR), which were predicted to be transcribed from the same strand. The gene arrangement pattern was identical to those of typical annelids. The overall nucleotides composition includes 35.01% A, 37.41% T, 12.65% G, and 14.93% C, with an AT bias of 72.42%. Genes overlap in a total of 23bp in six locations, with the longest (12 bp) located between ND2 and COX1. And, there were totally 201 bp of intergenic sequences which were distributed among 16 locations, ranging from 1–43 bp in size. Fifteen pairs of genes were directly adjacent without overlapping or intergenic nucleotides.

All the protein-coding genes started with ATG codon, except COX3 gene which employed TTG as start codon. Three different stop codons were used in *O*. *jantseanus*: TAA (ND5, ND4L, ND1, CYTB, ATP6), TAG (ND3) and T– (ND4, ND2, COX1, COX2, ATP8, COX3, ND6) which was common in the reported Hirudinea (Figueroa et al. 2016). The AT content of protein-coding genes ranged from 66.20% (COX3) to 78.34% (ATP8).

The total length of the 22 tRNA genes was 1441bp, varying from 62bp (tRNA^Lys^ and tRNA^Trp^) to 71bp (tRNA^Gln^). All of them could be fold into the conventional secondary structure. The 12S and 16S rRNA were 743bp and 1175bp in size, respectively. The NCR of Hirudinea was 78–636bp in length and flanked by genes tRNA^Arg^ and tRNA^His^. The variability of this region in length among Hirudinea could be attributed to the regulation of transcription and the control of DNA replication. Here, the NCR of *O*. *jantseanus* was 299 bp in length and 84.95% in AT content.

In order to infer the phylogenetic position of *O. jantseanus*, eleven Hirudinea species were retrieved from GenBank, and *Lumbricus terrestris* (U24570) was used as outgroup (see Figure 1 for details). *Ozobranchus jantseanus* clustered within Arhynchobdellida clade which was similar with previous analysis, indicating that the order Rhynchobdellia was not monophyletic and the proboscis was not a synapomorphy (Apakupakul et al. 1999).

**Figure 1.**
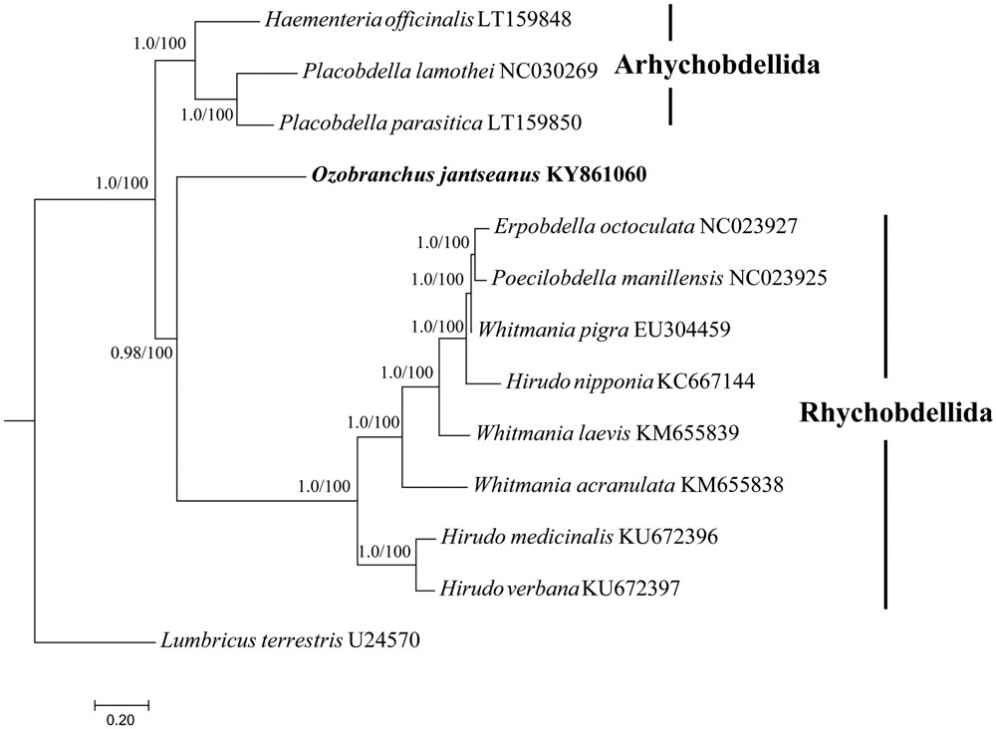
Phylogenetic analysis. Bayesian inference (BI) and Maximum likelihood (ML) method produced same topology based on the concatenated 13 PCGs, rooted at *Lumbricus terrestris* (U24570). Genbank accession numbers are given adjacent to species names. Numbers at nodes indicate posterior probabilities and bootstrap support values by BI and ML, respectively.
